# Sources of Low-Value Care Received by Medicare Beneficiaries and Associated Spending Within US Health Systems

**DOI:** 10.1001/jamanetworkopen.2023.33505

**Published:** 2023-09-20

**Authors:** Emma D. Chant, Maia Crawford, Ching-Wen Wendy Yang, Elliott S. Fisher, Nancy E. Morden, Ishani Ganguli

**Affiliations:** 1 Division of General Internal Medicine and Primary Care, Brigham and Women’s Hospital, Harvard Medical School, Boston, Massachusetts; 2 The Dartmouth Institute for Health Policy and Clinical Practice, Geisel School of Medicine at Dartmouth, Lebanon, New Hampshire; 3 Harvard Medical School, Boston, Massachusetts

## Abstract

This cross-sectional study examines referrals for low-value health care services and associated spending by ordering clinician among Medicare beneficiaries.

## Introduction

Low-value care—medical services with little or no net benefit in specific scenarios—harms patients and challenges health systems increasingly held accountable for care quality and spending. For all health systems, understanding sources of in-system low-value care (ie, which clinicians are responsible, which may vary by service [eg, due to different scopes of practice and care settings]) would facilitate efforts to reduce such care within their walls, where system leaders have greatest influence.^1^ In this cross-sectional analysis of Medicare beneficiaries attributed to US health systems, we assessed volume of and spending on 40 low-value services within these systems, including which clinician types ordered or referred for the services.

## Methods

We used 2016 to 2018 Medicare fee-for-service (FFS) administrative data for beneficiaries aged 65 years or older who were continuously enrolled in Medicare through 2018 or until death and attributed to 1 of 595 US health systems using Centers for Medicare and Medicaid Services Medicare Shared Savings Program methodology (eMethods in Supplement 1). We examined 40 low-value services relevant to older adults as identified by recommendations from the Choosing Wisely campaign and professional societies and operationalized in claims-based definitions from the Health Waste Calculator version 8.0 (Milliman MedInsight) and prior research (eTable in Supplement 1).^2,3^ For each service, we calculated the volume of services received by system-attributed beneficiaries in 2017-2018 that was ordered or referred for by in-system clinicians (affiliated with given beneficiaries’ systems based on the OneKey database [IQVIA]), including patients’ attributed primary care physicians (PCPs), other PCPs, advanced practice clinicians (APCs), and specialist physicians. We calculated Medicare spending on these services using narrow (claim line) and broad (claim case) definitions.

This study followed the Strengthening the Reporting of Observational Studies in Epidemiology (STROBE) reporting guideline and was approved by Dartmouth’s institutional review board; informed consent was not required because data were deidentified. Analyses performed March 2022 through May 2023 using SAS version 9.4 (SAS Institute) and Stata version 17.0 (StataCorp).

## Results

We studied 10.9 million beneficiaries (mean [SD] age, 74.6 [7.4] years; 6.3 million female [58%]) who received 8.4 million low-value services, of which 4.9 million (59%) were in-system. Among in-system services, 2.2 million (45%) originated from attributed PCPs (range by service type: 2%-70%), 713 700 (14%) from other PCPs (0%-43%), 179 094 (4%) from APCs (0%-11%), and 1.8 million (37%) from specialists (6%-93%) (Figure).

**Figure.**
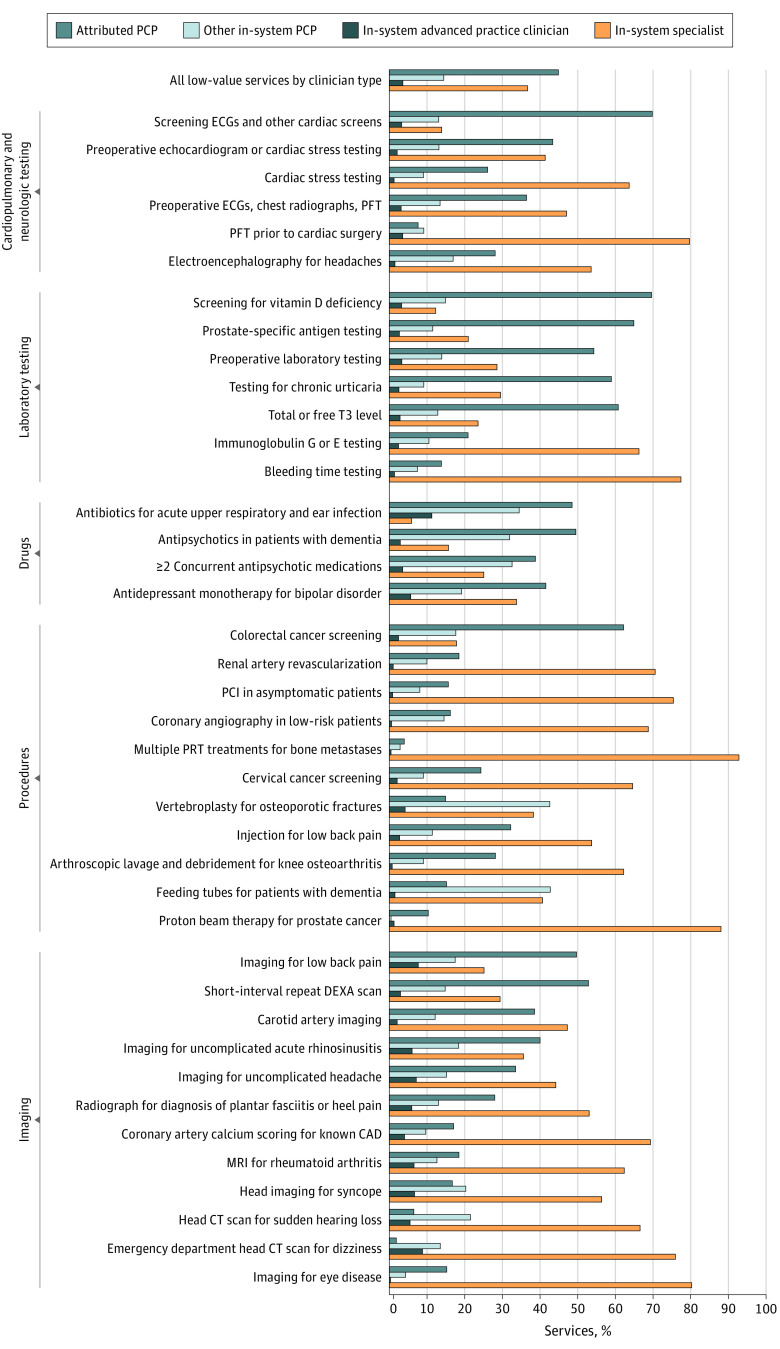
Distribution of Low-Value Services Ordered by Health System-Attributed Clinicians, by Clinician Type We ascribed each low-value service to its ordering clinician by using the National Provider Identifier (NPI) on the claim as follows: referring NPI (for nondrug services identifiable using the professional claims file), attending NPI (for nondrug inpatient and outpatient facility claims), or prescribing NPI (for drugs). We defined primary care physicians (PCPs) by specialty in general practice, family practice, internal medicine, pediatric medicine, or geriatric medicine. We defined specialist physicians as those with any other specialty. Advanced practice clinicians included nurse practitioners, certified clinical nurse specialists, and physician assistants. CAD indicates coronary artery disease; CT, computed tomography; DEXA, dual energy x-ray absorptiometry; ECG, electrocardiogram; MRI, magnetic resonance imaging; PCI, percutaneous coronary intervention; PFT, pulmonary function test; PRT, palliative radiation therapy.

Specialists ordered or referred for most examined imaging services and procedures, including coronary artery calcium scoring (69%) and renal artery revascularization (71%). PCPs ordered most of the examined laboratory tests and medications, including PSA testing (77%) and antipsychotic medications for patients with dementia (81%). Patients often received antibiotics for acute upper respiratory tract infections or ear infections from other PCPs (34%) or APCs (11%).

In-system spending was $347 296 813 by the narrow, claim-line definition and $1 085 086 079 by the broad definition. Of the narrow spending total, $100 861 220 (29.0%) originated from attributed PCPs, $40 186 844 (11.6%) from other PCPs, $7 350 743 (2.1%) from APCs, and $198 898 005 (57.3%) from specialists (Table). Specialists’ share of spending was higher than their share of service volume for 28 of 40 examined services.

**Table.** Medicare Spending on Low-Value Services and Distribution by Ordering Clinician Type

Low-value services	Spending definition, $^a^		In-system Medicare spending (narrow definition) on services, $ (%)^b^			
	Broad	Narrow	Attributed PCP	Other PCP	APC	Specialist
All services	1 085 086 079	347 296 813	100 861 220 (29.0)	40 186 844 (11.6)	7 350 743 (2.1)	198 898 005 (57.3)
Cardiopulmonary and neurologic testing						
Screening electrocardiograms and other cardiac screens	78 424 624	12 801 564	9 151 006 (71.5)	1 721 386 (13.4)	422 963 (3.3)	1 506 209 (11.8)
Preoperative echo or cardiac stress testing	803 527	633 813	267 519 (42.2)	91 231 (14.4)	20 107 (3.2)	254 956 (40.2)
Cardiac stress testing	45 087 683	36 718 036	8 866 728 (24.1)	3 210 219 (8.7)	471 592 (1.3)	24 169 498 (65.8)
Preoperative electrocardiograms, chest radiographs, or PFT	18 782 721	1 577 266	445 361 (28.2)	181 390 (11.5)	50 481 (3.2)	900 034 (57.1)
PFT prior to cardiac surgery	732 464	19 269	1899 (9.9)	2349 (12.2)	730 (3.8)	14 290 (74.2)
Electroencephalography for headaches	1 510 209	942 947	298 816 (31.7)	119 948 (12.7)	11 410 (1.2)	512 772 (54.4)
Laboratory testing						
Screening for vitamin D deficiency	24 168 964	4 568 709	3 192 634 (69.9)	663 716 (14.5)	164 901 (3.6)	547 459 (12.0)
Prostate-specific antigen testing	72 186 989	17 744 614	11 043 838 (62.2)	2 020 579 (11.4)	493 160 (2.8)	4 187 036 (23.6)
Preoperative laboratory testing	64 946 592	5 424 324	3 242 637 (59.8)	861 652 (15.9)	219 273 (4.0)	1 100 762 (20.3)
Testing for chronic urticaria	42 906	8403	5384 (64.1)	572 (6.8)	NR^c^	2447 (29.1)
Total or free T3 level	9 219 835	1 183 365	722 609 (61.1)	148 028 (12.5)	33 950 (2.9)	278 778 (23.6)
Immunoglobulin G or E testing	2 221 730	205 665	40 430 (19.7)	21 691 (10.5)	4397 (2.1)	139 147 (67.7)
Bleeding time testing	639 455	5081	849 (16.7)	291 (5.7)	NR^c^	3942 (77.6)
Drugs						
Antibiotics for acute upper respiratory and ear infections	2 394 325	2 394 325	1 099 774 (45.9)	757 955 (31.7)	251 151 (10.5)	285 445 (11.9)
Antipsychotics in patients with dementia	15 817 613	15 817 613	5 240 865 (33.1)	3 505 573 (22.2)	287 448 (1.8)	6 783 727 (42.9)
≥2 concurrent antipsychotic medications	24 867 264	24 867 264	7 717 480 (31.0)	7 869 852 (31.6)	874 802 (3.5)	8 405 129 (33.8)
Antidepressant monotherapy for bipolar disorder	125 717	125 717	22 186 (17.6)	21 118 (16.8)	6285 (5.0)	76 128 (60.6)
Procedures						
Colorectal cancer screening	18 136 059	7 571 769	3 440 144 (45.4)	1 319 821 (17.4)	117 183 (1.5)	2 694 622 (35.6)
Renal artery revascularization	7 576 408	6 723 430	1 304 296 (19.4)	284 067 (4.2)	60 315 (0.9)	5 074 752 (75.5)
PCI in asymptomatic patients	65 850 681	64 053 815	10 135 817 (15.8)	4 245 108 (6.6)	548 476 (0.9)	49 124 414 (76.7)
Coronary angiography in low-risk patients	11 338 155	7 872 849	1 428 341 (18.1)	672 383 (8.5)	53 674 (0.7)	5 718 452 (72.6)
Multiple PRT treatments for bone metastases	392 631	270 524	5987 (2.2)	NR^c^	NR^c^	264 536 (97.8)
Cervical cancer screening	26 369 239	14 090 862	3 188 627 (22.6)	1 182 323 (8.4)	246 867 (1.8)	9 473 045 (67.2)
Vertebroplasty for osteoporotic fractures	7 871 295	6 469 155	1 462 394 (22.6)	977 072 (15.1)	540 553 (8.4)	3 489 137 (53.9)
Injection for low back pain	47 532 442	42 441 032	11 887 442 (28.0)	4 277 403 (10.1)	1 116 571 (2.6)	25 159 616 (59.3)
Arthroscopic lavage and debridement for knee osteoarthritis	585 647	585 647	136 352 (23.3)	45 261 (7.7)	NR^c^	404 034 (69.0)
Feeding tubes for patients with dementia	1 534 398	1 162 378	237 548 (20.4)	349 746 (30.1)	23 099 (2.0)	551 986 (47.5)
Proton beam therapy for prostate cancer	2 103 732	1 953 030	42 433 (2.2)	22 957 (1.2)	NR^c^	1 887 639 (96.7)
Imaging						
Imaging for low back pain	1 715 669	903 789	438 504 (48.5)	134 427 (14.9)	48 079 (5.3)	282 779 (31.3)
Short-interval repeat DEXA scan	6 033 127	3 039 017	1 648 980 (54.3)	445 569 (14.7)	92 974 (3.1)	851 494 (28.0)
Carotid artery imaging	22 811 459	13 525 612	5 142 684 (38.0)	1 584 048 (11.7)	279 071 (2.1)	6 519 808 (48.2)
Imaging for uncomplicated acute rhinosinusitis	3 833 549	1 320 430	338 068 (25.6)	205 987 (15.6)	100 567 (7.6)	675 808 (51.2)
Imaging for uncomplicated headache	11 098 304	5 041 998	1 812 817 (36.0)	735 108 (14.6)	318 232 (6.3)	2 175 842 (43.2)
Radiograph for diagnosis of plantar fasciitis or heel pain	907 409	316 140	93 068 (29.4)	39 210 (12.4)	16 629 (5.3)	167 233 (52.9)
Coronary artery calcium scoring for known CAD	440 423	386 547	35 020 (9.1)	41 022 (10.6)	27 391 (7.1)	283 113 (73.2)
MRI for rheumatoid arthritis	149 872	126 993	25 597 (20.2)	14 360 (11.3)	7132 (5.6)	79 904 (62.9)
Head imaging for syncope	8 644 773	1 378 310	321 968 (23.4)	241 249 (17.5)	91 083 (6.6)	724 010 (52.5)
Head CT scan for sudden hearing loss	3 044 736	919 105	89 531 (9.7)	105 950 (11.5)	49 554 (5.4)	674 070 (73.3)
Emergency department head CT scan for dizziness	12 729 758	2 360 101	52 335 (2.2)	312 218 (13.2)	214 635 (9.1)	1 780 914 (75.5)
Imaging for eye disease	462 413 694	39 746 305	6 233 252 (15.7)	1 754 007 (4.4)	86 007 (0.2)	31 673 039 (79.7)

Abbreviations: APC, advanced practice clinician; CAD, coronary artery disease; CT, computed tomography; DEXA, dual energy x-ray absorptiometry; MRI, magnetic resonance imaging; NPI, National Provider Identifier; NR, not reported; PCI, percutaneous coronary intervention; PCP, primary care physician; PFT, pulmonary function test; PRT, palliative radiation therapy.

^a^ Narrow and broad definitions are given to provide a range of calculated Medicare spending. For the narrow (claim line) definition, we counted only payments associated with a claim line identified as low value. For the broad (claim case) definition, we included the entire claim payment if a component claim line was identified as low value. Spending could not be calculated for nondrug services provided in inpatient and skilled nursing facility settings because individual service charges are not itemized on these claims. Six service categories had more than 5% of services excluded when calculating total Medicare spending: renal artery revascularization (10.9% excluded), percutaneous artery intervention in asymptomatic patients (16.0%), coronary angiography in low-risk patients (19.5%), vertebroplasty for osteoporotic fractures (57.9%) and feeding tubes for patients with dementia (52.8%).

^b^ We ascribed each low-value service to its ordering clinician by using the NPI on the claim as follows: referring NPI (for nondrug services identifiable using the professional claims file), attending NPI (for nondrug inpatient and outpatient facility claims), or prescribing NPI (for drugs). We defined PCPs by specialty in general practice, family practice, internal medicine, pediatric medicine, or geriatric medicine. We defined specialist physicians as those with any other specialty. Advanced practice clinicians included nurse practitioners, certified clinical nurse specialists, and physician assistants.

^c^ Due to few services, counts are suppressed following data use guidelines.

## Discussion

In this national analysis, specialists accounted for a higher share of spending relative to volume across 40 low-value services, building on evidence that specialists have greater aggregate low-value spending^4^ to suggest they may both offer higher-cost services (eg, procedures) and use higher cost options within given low-value service definitions. PCPs ordered most examined drugs and laboratory tests, which have lower direct costs but potential for direct harms and care cascades.^5^ To encourage employed and affiliated clinicians to reduce these services, health systems could use evidence-based interventions including clinical decision support (eg, point-of-care alerts) and clinician feedback (eg, peer comparisons).^1^

Other in-system PCPs and APCs ordered a large share of low-value antibiotics for upper respiratory tract infections, likely during urgent care visits. To the extent that continuous clinician relationships decrease low-value care,^6^ systems might enable timely access to one’s own PCP, eg, through after-hours and telemedicine capacity and technology-enabled improvements in scheduling efficiency and flexibility.

Limitations of our study included limited generalizability beyond older FFS Medicare beneficiaries, possible misattribution due to billing errors or inconsistencies, that our findings were sensitive to the services included, and that clinicians ordering or referring for services may not have been primary decision-makers.

Direct spending on low-value services likely accounts for a small share of total Medicare spending,^3^ yet cascading costs and other harms compound. Understanding the scope and sources of within-system low-value care among attributed patients can inform targeted approaches to reduce such care, which may contribute materially to health systems’ success while improving patient outcomes.

## Notes

Supplement 1.
eMethods.
**eTable.** Low-Value Measure Definitions
eReferences.

Supplement 2.
Data Sharing Statement
