# Supplementary material for: Sources of Low-Value Care Received by Medicare Beneficiaries and Associated Spending Within US Health Systems
Source: JAMA Netw Open. 2023 Sep 20;6(9):e2333505. doi: 10.1001/jamanetworkopen.2023.33505 (PMC10512103; doi:10.1001/jamanetworkopen.2023.33505)
Supplement: Supplement 1. — eMethods. eTable. Low-Value Measure Definitions eReferences. [file jamanetwopen-e2333505-s001.pdf]

Supplemental Online Content

Chant ED, Crawford M, Yang CW, Fisher ES, Morden NE, Ganguli I. Sources of low-value care received by Medicare beneficiaries and associated spending within US health systems. *JAMA Netw Open.* 2023;6(9):e2333505.
doi:10.1001/jamanetworkopen.2023.33505

eMethods.

eTable. Low-Value Measure Definitions

eReferences.

This supplemental material has been provided by the authors to give readers additional information about their work.

© 2023 Chant ED et al. *JAMA Network Open.*

eMethods. Supplemental Methods
Adapted from Ganguli et al. (2021)[1]

Overview: We studied Medicare beneficiaries who were at least 65 years old as of January 1, 2016, had no hospice claims in 2017-2018, were continuously enrolled in Medicare through 2018 or until death, and were attributed to US health systems. For each of 40 low-value services, we determined the share of received services and associated Medicare spending originating from attributed primary care physicians (PCPs) and other in-system PCPs, advanced practice clinicians, and specialist physicians.

Data: We used 2016-2018 Medicare fee-for-service (FFS) administrative data from the Master Beneficiary Summary File (MBSF); 100% Parts A (inpatient) and B (outpatient) health plan claims (Files: Inpatient, MedPAR, Outpatient, Home Health Agency, Skilled Nursing Facility, and Durable Medical Equipment files); Part D (prescription) event file (random 40% sample); the Long Term Care Minimum Data Set; the Medicare Data on Provider Practice and Specialty (MD-PPAS) file; and FirstDataBank.[2]

Study Population: Our sample was comprised of US-residing beneficiaries ≥65 years old on January 1, 2016 and were continuously enrolled in Medicare Parts A and B through 2018 or until death. For measures using prescription data, we also required continuous Part D plan enrollment through 2018 or until death. We excluded members with any hospice claims in 2017-2018 (Long Term Care Minimum Data Set).

Beneficiary and Clinician Attribution to Health Systems: We used the 2018 AHRQ Compendium of US Health Systems to identify health systems.[3] We used the CMS Medicare Shared Savings Program attribution methodology as follows: attributed beneficiaries were attributed to the health system that provided the plurality of primary care services across 2017 and 2018 when possible; beneficiaries not receiving primary care services were attributed to a health system via specialist physician services.[4] We used the IQVIA OneKey database (which describes the relationships between providers, medical practices, and systems) to determine clinicians' health system affiliations.[5] We excluded systems with fewer than 250 attributed beneficiaries and pediatric health systems (based on a Compendium indicator).

Identification of Low-Value Services: We used 40 claims-based, low-value care definitions for services relevant to older adults, leveraging our prior work and the Milliman MedInsight Health Waste Calculator (version 8.0).[1,6] Non-Waste Calculator-based measures were antipsychotics for patients with dementia, percutaneous coronary intervention (PCI) for asymptomatic patients, injection for low back pain, feeding tubes for patients with dementia, and short-interval repeat dual-energy X-ray absorptiometry (DEXA) scan. The Milliman MedInsight Waste Calculator is a propriety, stand-alone software that uses International Classification of Diseases, Ninth and Tenth Revision (ICD-9 and ICD-10) diagnosis codes; American Medical Association procedural codes; and National Drug Code entries to assign health care services provided within specific clinical scenarios to one of three categories: not wasteful, likely wasteful, or wasteful. We defined low-value services conservatively in this work by only including those tagged as wasteful (i.e., we required the Calculator to flag with Degree of Certainty = "W" (Wasteful) to categorize them as low-value). We also required the Sufficient History flag = "Y" for all services except cervical cancer screening, prostate-specific antigen testing (PSA) testing, and screening electrocardiograms (ECGs), which had maximum lookback periods exceeding 1 year.

To define an incidence of a low value service, we counted multiple instances received by the same beneficiary but required a certain time difference between claim dates for discrete services (see eTable 1 for details) and counted only the first observed instance of service measurement occurring over a prolonged timeframe (feeding tubes for patients with dementia and long-term medications). We used 2016-2018 data to identify beneficiaries eligible for each service and to measure receipt of each low-value service among these beneficiaries in 2017-2018.

Physician Identification for Claims: Based this on consultation with experts in CMS clinical coding, we ascribed each low-value service to its ordering clinician using the NPI on the claim as follows: "referring NPI" (for non-drug services identifiable using the professional claims file), "attending NPI" (for non-drug inpatient and outpatient facility claims), or "prescribing NPI" (for drugs). For Waste Calculator-based measures, we identified the ordering clinician based on the National Provider Identifier (NPI) on the

“trigger” claim line flagged by the Waste Calculator as Wasteful. For non-Waste Calculator-based measures, we prioritized the referring NPI from the professional claim whenever possible.

To identify clinician type, we used the self-reported Provider Enrollment, Chain and Ownership System (PECOS) data (MD-PPAS file). PCPs were defined by specialty in general practice, family practice, internal medicine, pediatric medicine, or geriatric medicine. Specialist physicians were defined as those with any other specialty. Advanced practice clinicians included nurse practitioners, certified clinical nurse specialists, and physician assistants. The attributed PCP was the clinician providing the plurality of a patient’s primary care services in 2017-2018.

Analyses: We calculated the share of each service originating from patients’ attributed PCPs, other in-system PCPs, in-system advanced practice clinicians and in-system specialists. When calculating “All low-value services by clinician type,” we multiplied Part D-based measures (which used 40% sample data) by 2.5 to ensure comparability across measures.

We used narrow and broad definitions to calculate a range of in-system Medicare spending on these low-value services from 2017-2018. When reporting spending comparisons in the results (i.e., between clinician types and for total vs in-system spending), we used the narrow definition only. For the narrow (claim-line) definition, we counted only payments associated with a claim line identified as low-value. For the broad (claim case) definition, we included the entire claim payment if a component claim line was identified as low-value.[7] As charges are not itemized on claims for non-drug services provided in inpatient and skilled nursing facility settings, we excluded these claims from the spending calculations. We multiplied Part D-based measure estimates (which used 40% sample data) by 2.5 to ensure comparability across measures.

Following the Centers for Medicare and Medicaid Services data reporting rules, we suppressed results with 1-10 services and any values that would allow back-calculation of a result requiring suppression; when the latter occurred such that an additional cell required suppression, we chose the smallest cell to suppress. To construct the figure, we used a random number generator to assign values to suppressed results based on the difference between the unsuppressed row totals and the sum of the unsuppressed cells.

© 2023 Chant ED et al. *JAMA Network Open.*

eTable. Low-Value Measure Definitions

Measure	Eligible Beneficiaries	Flagged as low-value	Calculator -Based?	Counted Once per Beneficiary or Once per Service	Measure Description
Screening electrocardiograms (ECGs) and other cardiac screening[a]	All study beneficiaries	All trigger claims for eligible beneficiaries flagged by Calculator as "Wasteful" for MEASURE_ID AFP05	Yes	Once per service	Do not order annual ECGs or any other cardiac screening for low-risk patients without symptoms
Preoperative echocardiography or cardiac stress testing	All study beneficiaries with low or moderate risk surgery (Waste Calculator cohort flagged)	All trigger claims for eligible beneficiaries flagged by Calculator as "Wasteful" and Sufficient History flag="Y" for MEASURE_ID ASA02	Yes	Once per service	Do not perform baseline diagnostic cardiac testing or cardiac stress testing in asymptomatic stable patients with known cardiac disease undergoing low- or moderate-risk noncardiac surgery
Cardiac stress testing	All study beneficiaries	All trigger claims for eligible beneficiaries flagged by Calculator as "Wasteful" and Sufficient History flag="Y" for MEASURE_ID ACC00	Yes	Once per service	Do not perform stress cardiac imaging or advanced noninvasive imaging in the initial evaluation of patients without cardiac symptoms unless high-risk markers are present
Preoperative electrocardiograms (ECG), chest radiographs, or pulmonary function testing (PFT)	All study beneficiaries with low-risk surgery (Waste Calculator cohort flagged)	All trigger claims for eligible beneficiaries flagged by Calculator as "Wasteful" and Sufficient History flag="Y" for MEASURE_ID ASA01b	Yes	Once per service	Do not perform ECGs, chest radiographs, or PFT in patients without significant systemic disease (ASA I or II) undergoing low-risk surgery

© 2023 Chant ED et al. *JAMA Network Open.*

Measure	Eligible Beneficiaries	Flagged as low-value	Calculator -Based?	Counted Once per Beneficiary or Once per Service	Measure Description
Pulmonary function testing (PFT) prior to cardiac surgery	All study beneficiaries with cardiac surgery (Waste Calculator cohort flagged)	All trigger claims for eligible beneficiaries flagged by Calculator as "Wasteful" and Sufficient History flag="Y" for MEASURE_ID STHS05	Yes	Once per service	Do not recommend PFT prior to cardiac surgery in the absence of respiratory symptoms
Electroencephalography (EEG) for headaches	All study beneficiaries with diagnosis of headache (Waste Calculator cohort flagged)	All trigger claims for eligible beneficiaries flagged by Calculator as "Wasteful" and Sufficient History flag="Y" for MEASURE_ID ASA01b	Yes	Once per service	Do not perform EEG for headaches
Screening for vitamin D deficiency	All study beneficiaries; limited to 40% sample and continuous Part D enrollment during study period until death.	All trigger claims for eligible beneficiaries in denominator flagged by Calculator as "Wasteful" and Sufficient History flag="Y" for MEASURE_ID SCP01	Yes	Once per service	Do not perform population-based screening for 25-hydroxy-vitamin D deficiency
Prostate-specific antigen testing (PSA) testing[a]	All male study beneficiaries over 75 years of age	All trigger claims for eligible beneficiaries flagged by Calculator as "Wasteful" for MEASURE_ID URG01	Yes	Once per service	Do not perform PSA-based screening for prostate cancer in men older than 75 years
Preoperative laboratory testing	Study beneficiaries with low-risk surgery (cohort-flagged by Waste Calculator),<br><br>limited to 40% sample and continuous Part D enrollment during study period until death	All trigger claims for eligible beneficiaries flagged by Calculator as "Wasteful" and Sufficient History flag="Y" for MEASURE_ID ASA01a	Yes	Once per service	Do not perform baseline laboratory studies in patients without significant systemic disease (ASA I or II) undergoing low-risk surgery

© 2023 Chant ED et al. *JAMA Network Open.*

Measure	Eligible Beneficiaries	Flagged as low-value	Calculator -Based?	Counted Once per Beneficiary or Once per Service	Measure Description
Testing for chronic urticaria	All study beneficiaries w/ diagnosis of Urticaria (Waste Calculator cohort flagged)	All trigger claims for eligible beneficiaries flagged by Calculator as "Wasteful" and Sufficient History flag="Y" for MEASURE_ID AI03	Yes	Once per service	Do not routinely do diagnostic testing in patients with chronic urticaria
Total or free Triiodothyronine (T3) level	All study beneficiaries with diagnosis of hypothyroidism (Waste Calculator cohort flagged)	All trigger claims for eligible beneficiaries flagged by Calculator as "Wasteful" and Sufficient History flag="Y" for MEASURE_ID AACE04	Yes	Once per service	Do not order a total or free T3 level when assessing levothyroxine (T4) dose in hypothyroid patients
Immunoglobulin G or E testing	All study beneficiaries w/ diagnosis of Allergy (Waste Calculator cohort flagged)	All trigger claims for eligible beneficiaries flagged by Calculator as "Wasteful" and Sufficient History flag="Y" for MEASURE_ID AI02	Yes	Once per service	Do not perform unproven diagnostic tests, such as immunoglobulin G testing or an indiscriminate battery of immunoglobulin E tests, in the evaluation of allergy
Bleeding time testing	All study beneficiaries	All trigger claims for eligible beneficiaries flagged by Calculator as "Wasteful" and Sufficient History flag="Y" for MEASURE_ID SCP05	Yes	Once per service	Do not use bleeding time testing to guide patient care
Antibiotics for acute upper respiratory tract and ear infections	Study beneficiaries with diagnosis of upper respiratory infection or ear infection (Waste Calculator cohort flagged), limited to 40% sample and continuous Part D	All trigger claims for eligible beneficiaries in flagged by Calculator as "Wasteful" and Sufficient History flag="Y" for MEASURE_ID AP00	Yes	Once per service	Do not prescribe oral antibiotics for patients with upper respiratory tract or ear infection (acute sinusitis, viral respiratory illness, or acute otitis externa)

© 2023 Chant ED et al. *JAMA Network Open.*

**Measure**	**Eligible Beneficiaries**	**Flagged as low-value**	**Calculator -Based?**	**Counted Once per Beneficiary or Once per Service**	**Measure Description**
	enrollment during study period until death				
Antipsychotics for patients with dementia	Study beneficiaries with >=2 diagnoses of dementia >=7 days apart anytime in 2017- 2018. Exclude if beneficiary has had any diagnosis of severe mental illness in the same period. Limited to 40% sample and continuous Part D enrollment during study period until death.	Any antipsychotic prescription for eligible beneficiaries with a fill date after second dementia diagnosis	No	Once per beneficiary	Do not use antipsychotics as first choice to treat behavioral and psychological symptoms of dementia
Two or more concurrent antipsychotic medications	Study beneficiaries with any prescription fill for an antipsychotic medication (Waste Calculator NDC codes), limited to 40% sample and continuous Part D enrollment during study period until death.	All trigger claims for eligible beneficiaries flagged by Calculator as "Wasteful" and Sufficient History flag="Y" for MEASURE_ID APA01	Yes	Once per beneficiary	Do not routinely prescribe 2 or more antipsychotic medications concurrently
Antidepressant monotherapy for bipolar disorder	Study beneficiaries with diagnosis of bipolar disorder (Waste Calculator cohort flagged), limited to 40% sample and continuous Part D enrollment during study period until death and Part	All trigger claims for eligible beneficiaries flagged by Calculator as "Wasteful" and Sufficient History flag="Y" for MEASURE_ID DOR85	Yes	Once per beneficiary	Do not prescribe antidepressants as monotherapy for patients with bipolar I disorder

© 2023 Chant ED et al. *JAMA Network Open.*

Measure	Eligible Beneficiaries	Flagged as low-value	Calculator -Based?	Counted Once per Beneficiary or Once per Service	Measure Description
	D enrollment for all AB months.				
Colorectal cancer screening	All study beneficiaries	All trigger claims for eligible beneficiaries flagged by Calculator as "Wasteful" and Sufficient History flag="Y" for MEASURE_ID GE01	Yes	Once per service	Do not order unnecessary screening for colorectal cancer in adults older than 45 years
Renal artery revascularization	All study beneficiaries with diagnosis of renal artery stenosis (Waste Calculator cohort flagged)	All trigger claims for eligible beneficiaries flagged by Calculator as "Wasteful" and Sufficient History flag="Y" for MEASURE_ID DOR124	Yes	Once per service	Do not perform revascularization without prior medical management for renal artery stenosis
Percutaneous coronary intervention (PCI) for asymptomatic patients	Study beneficiaries with ischemic heart disease (defined by presence of Chronic Conditions Warehouse first indication date prior to December 31, 2018) and established diagnosis of acute myocardial infarction (defined by presence of CCW first indication date prior to July 1, 2018)	All claims for eligible beneficiaries with percutaneous coronary intervention procedure codes (coronary stent, balloon angioplasty, or atherectomy) >=6 months after CCW 1st indication date for both acute myocardial infarction and ischemic heart disease. Exclude beneficiary if the PCI was done during, or within 14 days after, an emergency department visit	No	Once per service (at least 7 days apart)	Avoid PCI for stable, asymptomatic patients with normal or only mildly abnormal adequate stress test results
Coronary angiography in low-risk patients	All study beneficiaries	All trigger claims for eligible beneficiaries flagged by Calculator as "Wasteful" and	Yes	Once per service	Do not perform coronary angiography in patients without cardiac symptoms

© 2023 Chant ED et al. *JAMA Network Open.*

Measure	Eligible Beneficiaries	Flagged as low-value	Calculator -Based?	Counted Once per Beneficiary or Once per Service	Measure Description
		Sufficient History flag="Y" for MEASURE_ID SNUC01			unless high-risk markers are present
Multiple palliative radiotherapy treatments for bone metastases	All study beneficiaries with diagnosis of bone metastases (Waste-calculator cohort flagged)	All trigger claims for eligible beneficiaries flagged by Calculator as "Wasteful" and Sufficient History flag="Y" for MEASURE_ID HPM03	Yes	Once per service	Do not recommend more than a single fraction of palliative radiotherapy for an uncomplicated painful bone metastasis
Cervical cancer screening[a]	All female study beneficiaries	All trigger claims for eligible beneficiaries flagged by Calculator as "Wasteful" for MEASURE_ID AFP00	Yes	Once per service	Do not order unnecessary cervical cancer screening (Papanicolaou test and human papillomavirus test) in all women who have had adequate prior screening and are not otherwise at high risk for cervical cancer
Vertebroplasty for osteoporotic fractures	All study beneficiaries with diagnosis of vertebral fracture (Waste-calculator cohort flagged)	All trigger claims for eligible beneficiaries flagged by Calculator as "Wasteful" and Sufficient History flag="Y" for MEASURE_ID DOR121	Yes	Once per service	Do not perform vertebroplasty for osteoporotic vertebral fractures
Injection for low back pain	Beneficiaries with 2 diagnoses of low back pain >=7 days apart. Exclude beneficiary if they have any diagnosis of radiculopathy in 2017-2018.	All claims for eligible beneficiaries with >=1 epidural, facet, trigger point injection claim for a diagnosis of low back pain without etanercept on the same claim. Injection claim must be on or after the	No	Once per service (if >= 7 days apart)	Do not provide outpatient epidural, facet, or trigger point spinal injections for low back pain

© 2023 Chant ED et al. *JAMA Network Open.*

Measure	Eligible Beneficiaries	Flagged as low-value	Calculator -Based?	Counted Once per Beneficiary or Once per Service	Measure Description
		second diagnosis of low back pain			
Arthroscopic lavage and debridement for knee osteoarthritis	All study beneficiaries with diagnosis of knee osteoarthritis (Waste Calculator cohort flagged)	All trigger claims for eligible beneficiaries flagged by Calculator as "Wasteful" and Sufficient History flag="Y" for MEASURE_ID DOR21	Yes	Once per service	Do not perform an arthroscopic knee surgery with lavage and/or debridement for knee osteoarthritis
Feeding tubes for patients with dementia	Study beneficiaries with >=2 diagnoses of dementia >=7 days apart and long-term nursing home residence (combined length of stay in nursing facility >90 days across 2017- 2018)	Claims with procedure code for feeding tube on claim after both second diagnosis of dementia and first day of institutionalization	No	Once per beneficiary	Do not recommend percutaneous feeding tubes for patients with advanced dementia
Proton beam therapy for prostate cancer	All study beneficiaries with diagnosis of prostate cancer (Waste Calculator cohort flagged)	All trigger claims for eligible beneficiaries flagged by Calculator as "Wasteful" and Sufficient History flag="Y" for MEASURE_ID ASRO04	Yes	Once per service	Do not routinely recommend proton beam therapy for prostate cancer outside of a prospective clinical trial or registry
Imaging for low back pain	Study beneficiaries with diagnosis of low back pain (Waste Calculator cohort flagged), limited to 40% sample and continuous Part D enrollment during study period until death	All trigger claims for eligible beneficiaries flagged by Calculator as "Wasteful" and Sufficient History flag="Y" for MEASURE_ID AFP02	Yes	Once per service	Do not perform imaging for low back pain within the first 6 weeks unless red flags are present

© 2023 Chant ED et al. *JAMA Network Open.*

Measure	Eligible Beneficiaries	Flagged as low-value	Calculator -Based?	Counted Once per Beneficiary or Once per Service	Measure Description
Short-interval repeat dual-energy x-ray absorptiometry (DEXA) scan	Female beneficiaries with at least one dual energy X-ray absorptiometry (DEXA) in 2018. Exclude beneficiary if they have fragility fracture in 2016-2018 on or prior to date of last cohort-defining DEXA, if they have a diagnosis of cancer in 2016-2018.	All repeat claims of DEXA within >30 to <730 days of another DEXA	No	Once per service	Do not routinely repeat DEXA scans more often than once every 2 years
Carotid artery imaging	All study beneficiaries	All trigger claims for eligible beneficiaries flagged by Calculator as "Wasteful" and Sufficient History flag="Y" for MEASURE_ID AN02	Yes	Once per service	Do not perform imaging of the carotid arteries with a normal neurological exam, except in specific circumstances
Imaging for uncomplicated acute rhinosinusitis	All study beneficiaries with diagnosis of uncomplicated acute rhinosinusitis (Waste Calculator cohort flagged)	All trigger claims for eligible beneficiaries flagged by Calculator as "Wasteful" and Sufficient History flag="Y" for MEASURE_ID AOHN04	Yes	Once per service	Do not routinely perform radiographic imaging for patients who meet diagnostic criteria for uncomplicated acute rhinosinusitis
Imaging for headache	All study beneficiaries with diagnosis of uncomplicated headache (Waste Calculator cohort flagged	All trigger claims for eligible beneficiaries flagged by Calculator as "Wasteful" and Sufficient History flag="Y" for MEASURE_ID ACR01	Yes	Once per service	Do not perform imaging for uncomplicated headache

© 2023 Chant ED et al. *JAMA Network Open.*

Measure	Eligible Beneficiaries	Flagged as low-value	Calculator -Based?	Counted Once per Beneficiary or Once per Service	Measure Description
X-Ray for diagnosis of plantar fasciitis/heel pain	All study beneficiaries with diagnosis of plantar fasciitis/heel pain (Waste Calculator cohort flagged)	All trigger claims for eligible beneficiaries flagged by Calculator as "Wasteful" and Sufficient History flag="Y" for MEASURE_ID ACOE03	Yes	Once per service	Do not routinely order X-ray for diagnosis of plantar fasciitis/heel pain in employees who stand or walk at work.
Coronary artery calcium scoring for known coronary artery disease (CAD)	All study beneficiaries with diagnosis of coronary artery disease (Waste Calculator cohort flagged)	All trigger claims for eligible beneficiaries flagged by Calculator as "Wasteful" and Sufficient History flag="Y" for MEASURE_ID SCCT01	Yes	Once per service	Do not use coronary artery calcium scoring for patients with known CAD (including stents and bypass grafts)
Magnetic resonance imaging (MRI) for rheumatoid arthritis	All study beneficiaries with diagnosis of rheumatoid arthritis (Waste Calculator cohort flagged)	All trigger claims for eligible beneficiaries flagged by Calculator as "Wasteful" and Sufficient History flag="Y" for MEASURE_ID ACRH03	Yes	Once per service	Do not perform MRI of the peripheral joints to routinely monitor inflammatory arthritis
Head imaging for syncope	All study beneficiaries with diagnosis of syncope (Waste Calculator cohort flagged)	All trigger claims for eligible beneficiaries flagged by Calculator as "Wasteful" and Sufficient History flag="Y" for MEASURE_ID ACPY01	Yes	Once per service	Do not obtain brain imaging studies (CT scans or MRI) in the evaluation of simple syncope and a normal neurologic examination
Head computed tomography (CT) scan for sudden hearing loss	All study beneficiaries with diagnosis of sudden hearing loss (Waste Calculator cohort flagged)	All trigger claims for eligible beneficiaries flagged by Calculator as "Wasteful" and Sufficient History flag="Y" for MEASURE_ID AOHN01	Yes	Once per service	Do not order CT scan of the head or brain for sudden hearing loss

© 2023 Chant ED et al. *JAMA Network Open.*

Measure	Eligible Beneficiaries	Flagged as low-value	Calculator-Based?	Counted Once per Beneficiary or Once per Service	Measure Description
Emergency department head computed tomography (CT) scan for dizziness	All study beneficiaries with diagnosis in Waste Calculator "Dizziness" code set (Waste Calculator cohort flagged)	All trigger claims for eligible beneficiaries flagged by Calculator as "Wasteful" and Sufficient History flag="Y" for MEASURE_ID JH001	Yes	Once per service	Do not perform routine head CT scans for emergency department visits for dizziness
Imaging for eye disease	All study beneficiaries with diagnosis of eye disease (Waste Calculator cohort flagged)	All trigger claims for eligible beneficiaries flagged by Calculator as "Wasteful" and Sufficient History flag="Y" for MEASURE_ID AO02	Yes	Once per service	Do not routinely order imaging tests for patients without symptoms or signs of significant eye disease

[a] Sufficient history requirement waived for this measure.

© 2023 Chant ED et al. *JAMA Network Open.*

eReferences

1. Ganguli I, Morden NE, Yang CWW, Crawford M, Colla CH. Low-Value Care at the Actionable Level of Individual Health Systems. *JAMA Internal Medicine*. 2021;181(11):1490-1500. doi:10.1001/JAMAINTERNMED.2021.5531

2. Drug Database | Medication Decision Support. FDB (First Databank). Accessed May 8, 2023. https://www.fdbhealth.com/

3. Agency for Healthcare Research and Quality. Compendium of U.S. Health Systems, 2018. https://www.ahrq.gov/ chsp/data-resources/compendium-2016.html

4. *Medicare Shared Savings Program: Shared Savings and Losses and Assignment Methodology Specifications*.; 2017.

5. OneKey Healthcare Reference Data Set - United States - IQVIA. Accessed September 26, 2022. https://www.iqvia.com/locations/united-states/solutions/life-sciences/information-solutions/essential-information/onekey-reference-assets

6. Milliman. What Is the Health Waste Calculator?

7. Mafi JN, Reid RO, Baseman LH, et al. Trends in Low-Value Health Service Use and Spending in the US Medicare Fee-for-Service Program, 2014-2018. *JAMA Network Open*. 2021;4(2):e2037328. doi:10.1001/jamanetworkopen.2020.37328

© 2023 Chant ED et al. *JAMA Network Open.*
