# Supplementary material for: Sources of Low-Value Care Received by Medicare Beneficiaries and Associated Spending Within US Health Systems
Source: JAMA Netw Open. 2023 Sep 20;6(9):e2333505. doi: 10.1001/jamanetworkopen.2023.33505 (PMC10512103; doi:10.1001/jamanetworkopen.2023.33505)
Supplement: Supplement 2. — Data Sharing Statement [file jamanetwopen-e2333505-s002.pdf]

Data Sharing Statement

Chant. Sources of Low-Value Care Received by Medicare Beneficiaries and Associated Spending Within US Health Systems. *JAMA Netw Open*. Published September 13, 2023. doi:10.1001/jamanetworkopen.2023.33505

Data
Data available: No

Additional Information
Explanation for why data not available: Data sharing is restricted by Centers for Medicare and Medicaid Services data use agreement
